# Cultivation and detection of endophytic aerobic methanotrophs isolated from *Sphagnum* species as a perspective for environmental biotechnology

**DOI:** 10.1186/s13568-014-0058-3

**Published:** 2014-08-02

**Authors:** Zofia Stępniewska, Agnieszka Kuźniar

**Affiliations:** 1 Department of Biochemistry and Environmental Chemistry, The John Paul II Catholic University of Lublin, Konstantynow 1I, Lublin, 20-708, Poland

**Keywords:** Endophytic bacteria, Methanotrophs, Methane, Sphagum sp

## Abstract

Enriched cultures of microorganisms are an essential step in the production of inoculum of these organisms for biotechnology and bioengineering. The potential application of methanotrophic microorganisms for removal of methane produced from landfills and coal mines as well as biodegradation of toxic compounds has been widely studied. Therefore, searching for new sources of methanotrophs can contribute to increasing the possibilities of biotechnology and bioengineering.

Enrichment cultures of endophytic methanotrophs from *Sphagnum* sp. were initiated in NMS medium, a most widely used medium for cultivation of methanotrophic bacteria from various environments proposed in 1970 by Whittenbury. Incubation was carried out at 10, 20, 30, and 37°C with vigorous shaking on a shaker (180 rpm). The source of carbon and energy for endophytes were methane at the concentration range between 1-20%.

It appeared that the consortium of endophytic bacteria grew only at the temperature of 20 and 30°C. During the culture of endophytes, the measurements of gas concentration showed a steady loss of methane and oxygen, as well as accumulation of carbon dioxide as a CH_4_ oxidation product.

The use of FISH has made characterization of endophytic consortia possible. It turned out that the population of endophytes consists of type I and II methanotrophs as well as associated non-methanotrophic bacteria.

Furthermore, we determined the potential of the examined bacteria for methane oxidation, which ranged up to 4,7 μMCH_4_ per ml of the population of endophytes per day.

## Introduction

The history of the use of microorganisms by man is as old as the human civilization itself. Microorganisms have long served humans in industrial applications e.g. production of food, drug, and cosmetics. Recently, progress can be seen in the use of microorganisms for environmental biotechnology, namely removal of greenhouse gases from various sites (landfills, coal mines), biodegradation of toxic compounds, wastewater treatment etc. (Hamer [2010]). Each species of microorganisms found in the earth has its own specific requirements of nutrients and growth conditions that are often difficult to mimic in the culture media in the laboratory. Therefore, one of the most up-to-date trends in environmental biotechnology is seeking for new sources of microorganisms with a wide range of growth conditions and, especially, the possibility of utilizing waste material for cultivation (Minamisawa [2006]).

A unique and fascinating group of microorganisms are methanotrophic bacteria, which were discovered over a century ago and yet are still of great interest. Aerobic methanotrophic bacteria, which use methane as the sole source of carbon and energy, act as a major methane sink. Methanotrophic bacteria have been studied in soils, deserts, landfills, tundra, wetlands, rice paddies, sediments, lakes, and marine environments (Hanson and Hanson [1996]), as well as in the atmosphere (Santl-Temkiv et al. [2013]) and coal mines (Stępniewska et al. [2006]).

Methanotrophs have significant potential for applied microbiology and biochemical engineering, including bioremediation of pollutants (e.g. halogenated hydrocarbons) via co-metabolism by MMOs, biotransformation of diverse organic substrates (e.g., propylene to epoxypropane, production of chiral alcohols), assimilation of methane to mitigate greenhouse effects, and production of commercially relevant compounds (e.g., single cell protein, poly-_ hydroxybutyrate, astaxanthin). Therefore, engineering of methanotrophs is very important to their industrial applications. Recent years have seen significant progress in functional genomics and proteomics of methanotrophs (Hamer [2010]; Jiang et al. [2010]; Trotsenko and Murrell [2008]). Understanding of natural microbially mediated processes has been severely retarded by the common requirement to study microorganisms only as pure monocultures under aseptic conditions, in spite of the fact that, in all real environments, microbial strains function in a community (Hamer [2010]).

Based on basic research and deep knowledge of methanotrophs, there are more possibilities to make methanotrophs become important and universal industrial microorganisms (Jiang et al. [2010]). Therefore, these studies focused on recognition of the conditions of culturing aerobic endophytic methanotrophs and their application in environmental biotechnology and bioengineering.

The aim of our present study was to investigate a new perspective of the use of *Sphagnum* endophytic methanotrophs in environmental biotechnology.

## Material and methods

### Plant material

*Sphagnum* sp*.* materials were selected from Moszne peatbog (the Poleski National Park, Eastern Poland). The samples of the crops were collected in September 2011 from four different sites (meadow peat soil, bog forest).

### Isolation of endophytic methanotrophs

The endophytic bacteria were isolated from young *Sphagnum* sp. plants. Randomly selected plants were uprooted manually and washed in running tap water. Leaf sections of 1–2 cm length were excised using a flame-sterilized scalpel. The samples were blotted dry with filter paper; next, surface sterilization of shoot and root pieces was performed with the following immersion sequence: 70% ethanol for 1 min and 3% sodium hypochlorite for 5 min. Then, they were rinsed four times with sterile water and dried in laminar flow. The cut ends of surface sterilized segments were removed with a flame-sterilized scalpel and placed in appropriate NMS solid medium, i.e. a most widely used medium for cultivation of methanotrophic bacteria from various environments proposed in 1970 by Whittenbury, with the cut surface touching the medium. Due to the volatile nature of the essential metabolic substrates used by methanotrophs (CH_4_, O_2_), the cultures were grown in glass bottles with a capacity of 120 cm^3^, equipped with a hermetically closed cover, allowing application and collection of gases. The glass bottles were incubated for six to ten days at 30°C. The growth of methanotrophic bacteria was stimulated by supplying CH_4_ (10%v/v) for the cultivation. The proportion between the solid medium and gaseous culture was always 1:5. Subsequently, a single colony was transferred to 120 cm^3^ bottles containing liquid NMS medium and the cultures were incubated at 10°C, 20°C, and 30°C with 180 rpm shaking. Meanwhile, the concentration of bacterial cells in the suspension was determined spectrophotometrically by absorbance at 600 nm. Concurrently, methane consumption in the headspace was measured with a gas chromatograph equipped with a flame ionization detector, a thermal conductivity detector, and an electron capture detector (SIMADZU, GC 2010). Nitrogen and helium were used a carrier gas (30 mL min−1) and the injector, oven, and detector temperatures were 250°C (FID) and 150°C (TCD). The flame gases including H_2_ and compressed air were set at 20 and 30 mL min−1, respectively.

It has been demonstrated that in natural conditions methanotrophic bacteria cooperate with other microorganisms and their pure cultures are unstable for extended periods of time (Hoefman et al. [2010]). In these studies, we investigated a consortium of whole microbial communities.

### Morphology and cell shape

The isolated population of endophytic methanotrophs (actively growing) were collected by centrifugation, connected with 0,2% phosphotungstic acid in the ratio 1:1. Then, the mixtures were transferred onto a copper grid covered with a formvar film. After drying the grid, photographs of endophytic methanotrophs were taken with the use of electron microscopy techniques (LEO 912AB) with an electron energy filter. *Methylosinus trichosporium* and *Methylomonas methanica* originating from Russian Academy of Sciences, Institute of Biochemistry and Physiology of Microorganisms were used as a positive control.

### Identification of the endophytic bacterial population

#### DNA extraction

Total DNA was extracted from enrichment cultures where visual turbidity developed using the method described previously by Sambrook and Russell ([2001]) with some modifications. Cells from 10 ml samples of late-exponential cultures were collected by centrifugation. The pellet was suspended in 250μl of TE buffer containing 50mM Tris–HCl (pH=8.0) and 50mM EDTA (pH=8.0). To achieve complete lysis of the cells, 1 ml of GES buffer (pH=8.0) containing 5M guanidine thiocyanate, 100mM EDTA, and 0.5% sarkosyl was added. The mixture was incubated at room temperature for 10 minutes and then “crude lysates” were cooled on ice. After addition of 125μl of ammonium acetate (7.5M), the samples were mixed and further incubated on ice. The DNA obtained was purified with 250μl of a chloroform-isoamyl alcohol (24:1) mixture, precipitated with isopropanol, washed with cold ethanol, and dissolved in 50μl of sterile distilled water.

### PCR amplification

Specific primers for functional genes and 16S rRNA of the methanotrophic bacteria synthesized at GENOMED, Warsaw (Poland), were used. Polymerase chain reactions (PCR) were run in a programmable thermal cycler (MJmini, Bio-Rad). The reaction mixture (50μl) consisted of 1xPCR MIX: PCR amplification buffer, 0.05 U/μl Taq DNA polymerase, 0.4mM of each dNTP, 4mM MgCl_2_ (FERMENTAS), forward and reverse primers at 0.1mM and 3.5 μl of template DNA. The reaction conditions consisted of initial denaturation at 96°C for 4 minutes, 30 cycles of 94°C for 2 min, primer annealing at 56°C for type I and 55°C for type II each for 1 min, and elongation at 72°C for 1 min. Final elongation was performed at 72°C for 3 minutes. The amplification products were analyzed by electrophoresis in 1% agarose gel and stained with ethidium bromide.

The sequencing processes were performed on the purified product immediately after the PCR reaction in the Laboratory of DNA Sequencing and Oligonucleotide Synthesis (GENOMED, Warsaw, Poland). The sequences obtained were compared to the closest relatives in the GenBank database using the BLAST program.

All sequences have been deposited in GenBank under accession numbers: KJ657737 - KJ657745 and KJ623261, KJ 1713769.

### FISH

#### Fixation procedure

Cells growing in the logarithmic phase were harvested by centrifugation and resuspended in 0.5ml of phosphate-buffered saline (PBS). The suspensions were then mixed with 1ml of 4% (w/v) freshly prepared paraformaldehyde solution and fixed for 1 h at room temperature. The fixed cells were collected by centrifugation (6000•g for 1min) and washed twice with PBS to ensure removal of paraformaldehyde. The resulting pellet was resuspended in 0.3 ml of 99.9% (v/v) ethanol and stored at 20°C until use. For use in FISH, probe Mγ84: AGCCCGCGACTGCTCACC (type I), Mγ705: CTAGACTTCCTTGTGGTC (type I), and Mα450: CTATTACTGCCATGGACCTA (type II) was labeled with indocarbocyanine dye (Eller et al. [2001]). The oligonucleotide probes were synthesized and labeled with fluorochromes (CY3, CY5) by MWG Biotech (Ebersberg, Germany).

Hybridization was done on 75% ethanol-rinsed and dried slides with eight wells for independent positioning of the samples. Approximately 4μl of the fixed cell suspension was spread on each well, air dried, and dehydrated by successive passages through an ethanol series (50, 75, and 99.9% (v/v)) for 3 min each. A 50ml polypropylene Falcon tube containing a slip of filter paper soaked in hybridization buffer was used as a hybridization chamber, as described by Stahl and Amann [1991]. Hybridization buffer (10μl) containing 1M Tris–HCl (pH=8), 5M NaCl, 10% sodium dodecyl sulfate (SDS), and 20% formamide was placed on each spot of the fixed cells and then 1μl of fluorescent probe solution was added. The chamber was incubated for 60 min at the hybridization temperature. Then, the slides were washed at the hybridization temperature for 15 min in washing buffer (1M Tris–HCl, 0.5M EDTA, and 5M NaCl) and rinsed twice with distilled water. The slides were air dried, stained with 4,6- diamidino-2-phenylindole (DAPI; 2 M) for 10 min in the dark, rinsed again with distilled water, and finally air dried.

### Microscopy

The slides were analyzed by fluorescent microscope techniques (Nikon Eclipse 80i). The pictures were taken with a Digital Sight camera (Nikon) and processed using software program.

## Results

### Culture growth

**Figure 1:**
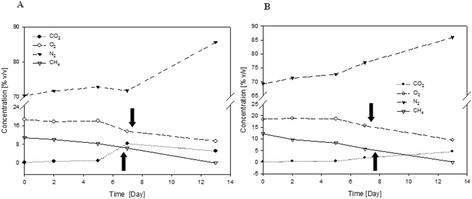
The dynamics of gases during the growth of the methanotrophic community, at 30oC (A) and 20oC (B) under 10% CH4 in the headspace.

It appeared that the consortium of endophytic bacteria grew at the temperature of 20 and 30°C, but not at 10°C and 37°C (Figure 1). A steady loss of methane and oxygen was shown, as well as accumulation of carbon dioxide as a CH_4_ oxidation product according to the equation:

(1) $$CH_{4}\;+\;2O_{2}\underset{\to }{\mathrm{MMO}}\;CO_{2}+\;H_{2}O\;+\;780\;\mathrm{kJ}\;\mathrm{mo}l^{‐1}\;+\;\mathrm{biomass}$$

**Figure 2:**
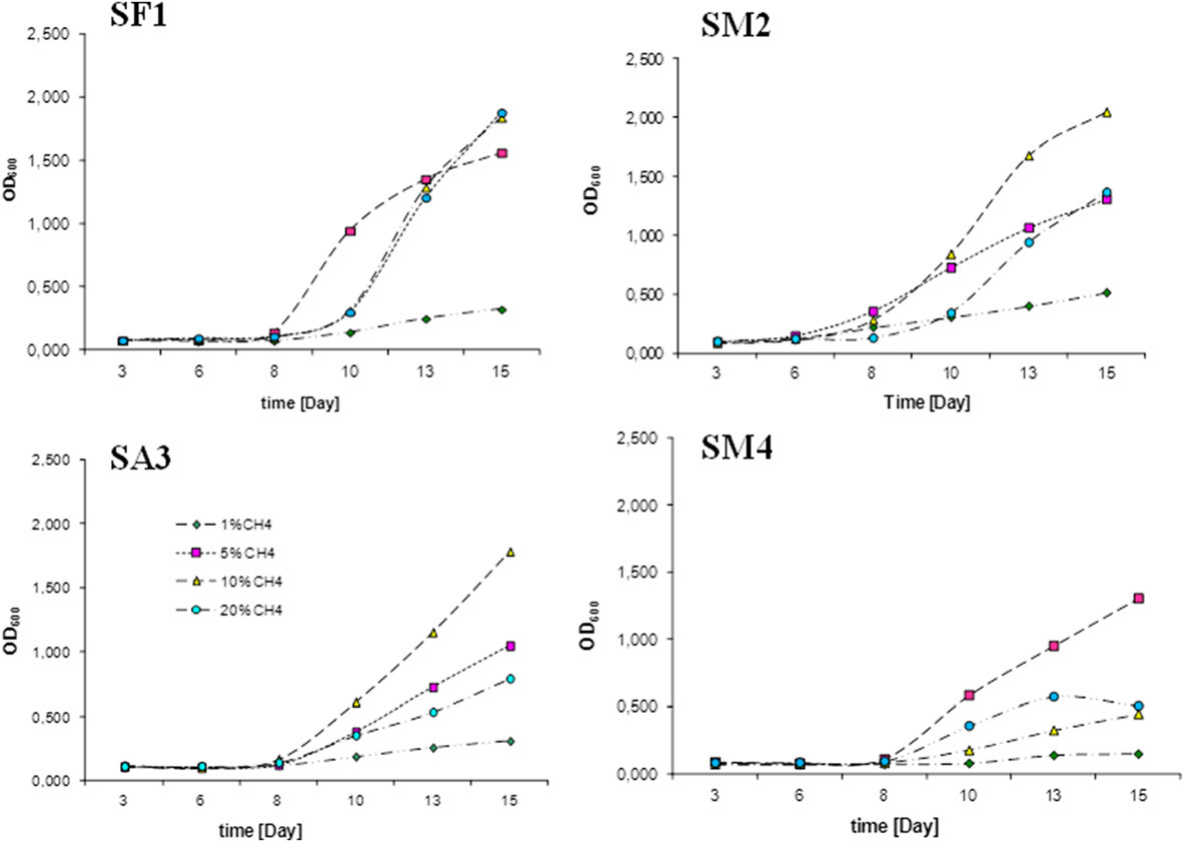
**The growth curves of methanotrophic consortium cultures (at different 1-20% concentrations of CH4) isolated from leaves of** ***Sphagnum*** **sp. gametophytes: SF1 –*S. flexuosum*, SM2–*S. magellanicum*** **2, SA3–*S. fallax*, SM4 –*S. magellanicum*** **4.**

During the initial phase, adaptation of endophytes ranged from 5 to 8 days at 30°C and up to 12 days at 20°C, which can be due to cellular metabolism at lower temperature (Figure 2). Then, there was rapid growth of the endophytic population (logarithmic phase), which was reflected by an increase in optical density in the range from 0.3 to 2.0 at 30°C and from 0.25 to 1.4 at 20°C, depending on the methane concentration (1-20%). After the logarithmic phase, lack of carbon/energy sources was observed and an increase in the concentration of waste products to a harmful level for methanotrophic consortia (about day 13, Figure 2). This time was referred to as the stationary phase of the enrichment cultures.

### Methanotrophic activity of the endophytic population

**Figure 3:**
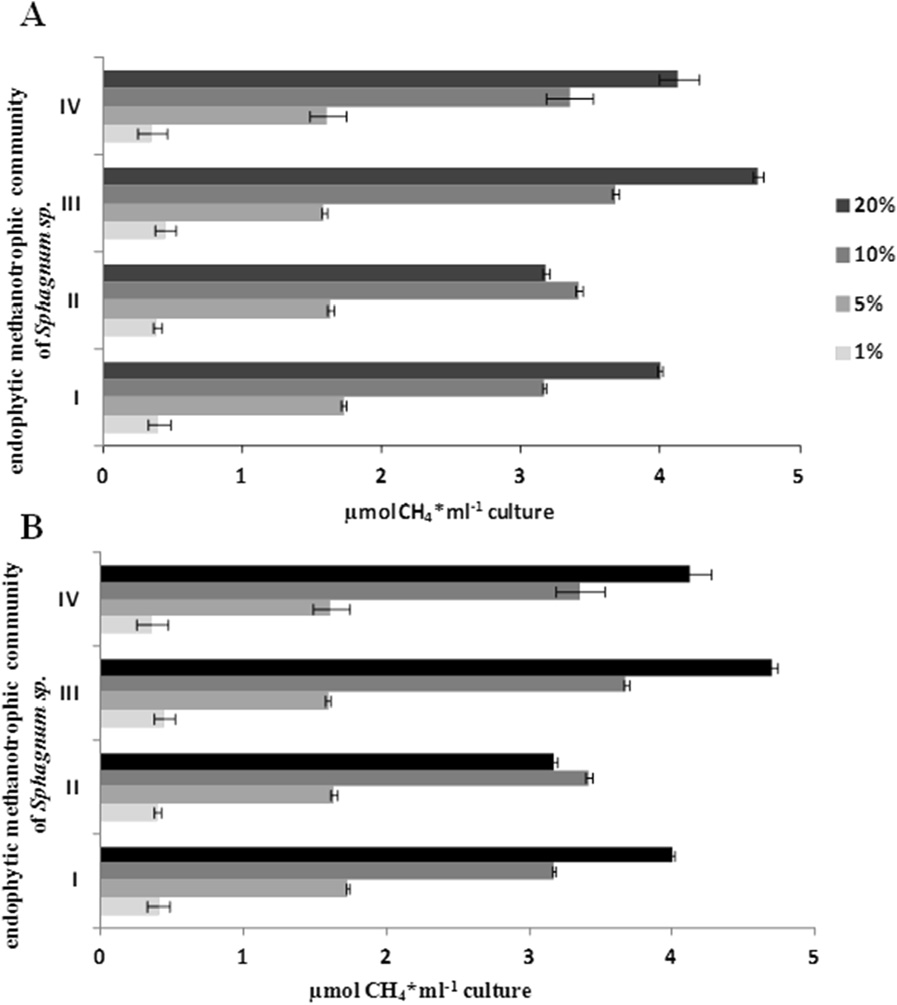
**Methanotrophic activity (MA) of the endophytic metanotrophic population obtained from leaves of*Sphagnum*sp gametophyte determined at 20oC (A) and 30oC (B) under different concentrations of methane (1- 20%).** I – *S. flexuosum* M1, II – *S. magellanicum* M2, III – *S. fallax* M3, IV – *S. magellanicum* M4.

The CH_4_ consumption in the enrichment culture (at 20 and 30°C) from the four localizations of *Sphagnum* sp. showed a clear decrease in the CH_4_ concentration tested in the headspace after 12–13 days of incubation (Figure 1). The highest CH_4_ consumption, below 20% of the initial CH_4_ concentration, was found in the isolated population from *Sphagnum magellanicum* (4,7 μMCH_4_ per ml liquid culture per day, Figure 3) at 30°C. No significant correlation in methanotrophic activity between the 20 and 30°C temperatures (UMW, p=0.89) was observed and no statistically significant differences between the activity of methanotrophic bacteria from the different species of *Sphagnum* sp. (UMW, p=0.85) were confirmed. A linear relationship with a high correlation coefficient (R^2^=0.99) between the methanotrophic activity of *Sphagnum* sp. endophytes and the methane concentration (in the range of CH_4_ from 0 to 10%, Table 1) was shown. The higher concentration of methane (over 10%) did not increase the methanotrophic activity of endophytes isolated from *Sphagnum* sp.. Furthermore, at a concentration of 20% CH_4_ it was observed to be even slightly lower, as in the case of the *S. magellanicum* M2 endophytes. The results obtained suggest that at 10% of methane complete saturation of the methane monooxygenase active center took place.

**Table 1:** Influence of the methane concentration (x) on the methanotrophic activity of endophytes (MA, y) determined at 20°C and 30°C (n = 8)

**Population**	**AM (**μ**MCH**_**4**_**ml**^**−1**^**liquid culture day**^**−1**^**)**		**R**^**2**^	
	20°C	30°C	20°C	30°C
SF1	y=0.3063x+0.1316	y=0.3063×+0.1315	0.99	0.99
SM2	y=0.337x+0.0147	y=0.337x+0.0147	0.99	0.99
SA3	y=0.3618x+0.0268	y=0.3618x+0.0268	0.99	0.99
SM4	y=0.3336x+0.0088	y=0.3336x+0.0089	0.99	0.99

### Characterization of the endophytic isolates

**Figure 4:**
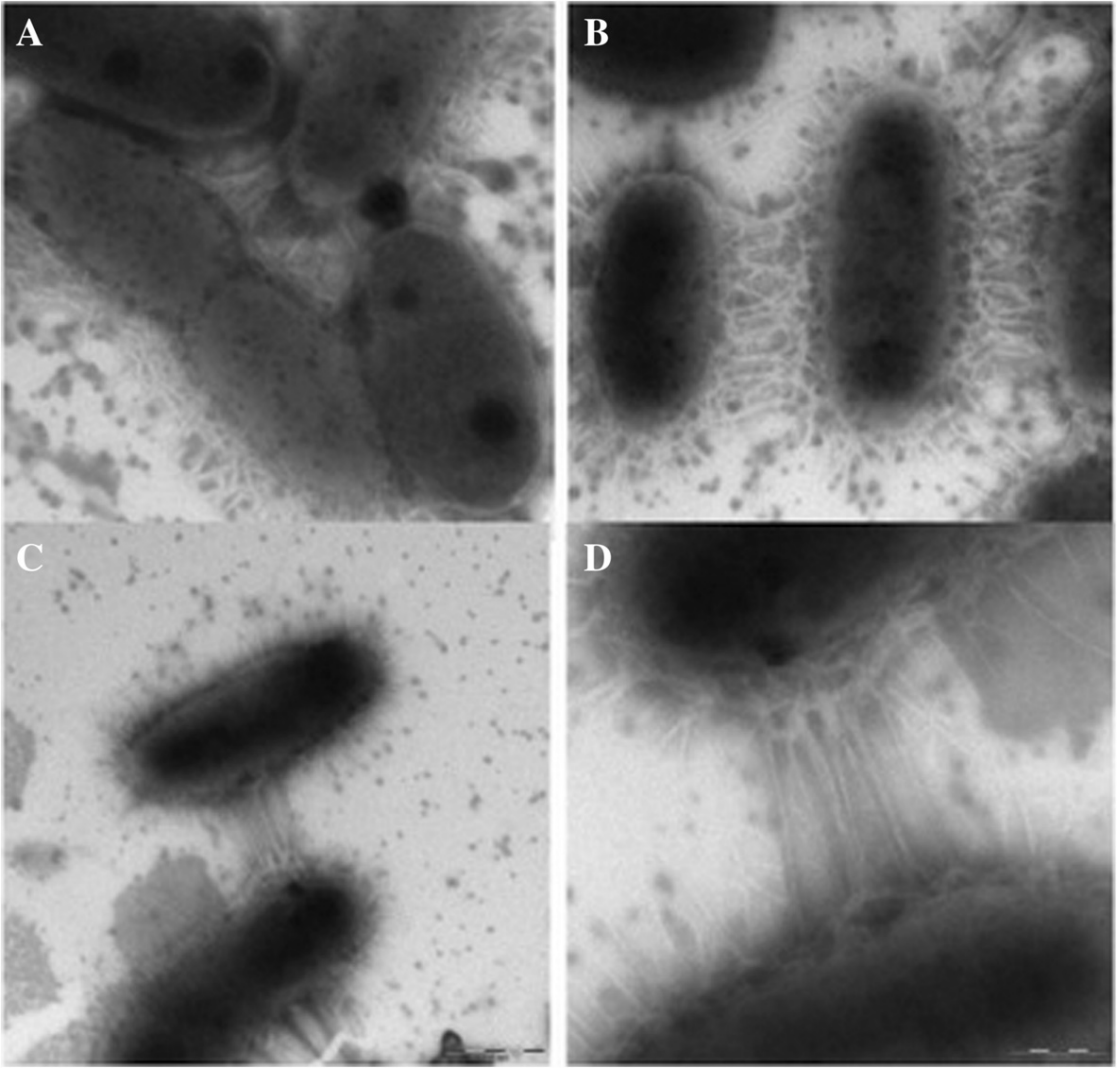
**Transmission electron micrograph of the methanotrophic community grown on the NMS medium with visible nanopod structure connecting neighboring bacteria**.

Transmission electron microscopy (TEM) showed that the SM2 and SM4 populations consisted of single coccoid cells that showed a cell wall typical of Gram-negative bacteria. In addition, cells from all the tested endophyte populations had a rod shape. SM2 and SA3 isolates exhibited a motile character. TEM microscopy (negatively stained cells) indicated that the cells of all the tested populations had extracellular structures called nanopods with a length of ca. 480 nm (Figure 4, Table 2).

**Table 2:** **Microscopic characterization of the methanotroph community isolated from different** ***Sphagnum*** **species**

**Name of isolates**	**Length/Width [**μ**m]**	**Cell shape**		**Nanopods**	**Flagella**
		**Cocco-bacilli**	**Rods**		
SF1	1,89×0,32	-	+	+	-
SM2	1,99×0,52	+	+	+	+
	φ=0,94				
SFA3	1,89×1,01	-	+	+	+
	2,72×0,84				
	1,65×0,76				
SM4	3,69×2,22	+	+	+	-
	3,47×0,65				
	φ=1,09				

### Fluorescence in situ hybridization

**Figure 5:**
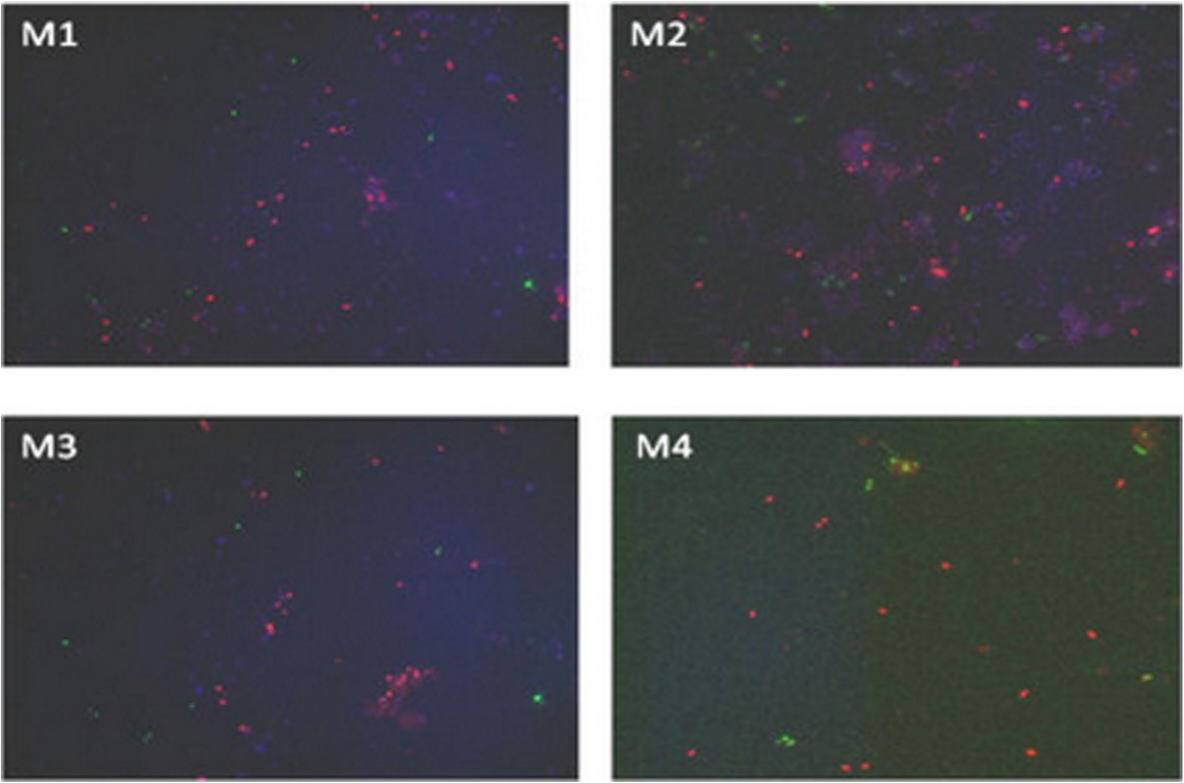
**Whole-cell specific hybridization of the endophytic metanotrophic population with probes: Mg84 (type I, pink), Ma 450 + Mg705 (type II, green) and DAPI staining (bacteria, dark blue) by FISH.** M1 – *S. flexuosum* , M2– *S. magellanicum* , M3– *S. fallax* , M4 – *S. magellanicum.*

The endophytic consortia were characterized with the use of FISH (**F**luorescence **i**n **s**itu **H**ybridization**)** analysis. The cultured population of endophytic microorganisms consisted of type I, which were the dominant population (pink cells), and type II methanotrophs (green cells), as well as associated non-methanotrophic bacteria (dark blue cells, Figure 5).

### Identification of the isolated endophytes

The methanotrophic communities isolated from *Sphagnum* mosses were investigated with use of general taxonomic markers like 16S rRNA, specific for type I and II methanotrophs. The 16S rRNA PCR products obtained (after purification, type I – 700 bp, type II – 530 bp) were sequenced using genomic DNA from *Sphagnum* mosses as a template. Then similarity of the tested sequences to nucleotide sequences from the GenBank database was assessed.

The identification results revealed the presence of both type I and type II methanotrophs. Among cultivable methanotrophs type I, there were different strains of the genus *Methylomonas*, whereas methanotrophs type II were represented by cultured strains belonging to the genera *Methylocystis* and *Methylosinus*. Molecular identification with the use of BLAST revealed high similarity of the methanotrophs cultured on the NMS medium to the genus *Methylocystis* (GenBank:. KJ623261, KJ657737, KJ657742) in the population isolated from *Sphagnum flexuosum* (99%), *Methylomonas* (GenBank: KJ657738, KJ657739, KJ657740, KJ657743) *Methylocystis* (GenBank: KJ 1713769) in the community isolated from *S. magellanicum* (94-100%), and to the genera *Methylocystis*, and *Methylosinus* (GenBank: KJ657744, KJ657745) in the population isolated from *S. fallax* (96-98%, Table 3).

**Table 3:** **Identification of cultured endophytic methanotrophs isolated from different** ***Sphagnum*** **species based on molecular genetic analysis**

**Isolate**	**Identity**	**Plant species**	**Max score**	**Max iden**	**Accession number (GenBank)**
SF1	*Methylocystis. echinoides*	*S. flexuosum*	850	99%	AJ4558502.1
	*Methylocystis sp.* DWT		850	99%	AJ868423.1
	*Methylomonas* sp. LC1		588	85%	DQ119049.1
SM2	*Methylomonas sp.* R-45373	*S. magellanicum* 2	1153	100%	FR798959.1
	*Methylocystis sp.* B3		863	99%	DQ119049.1
	*Methylomonas sp.* LW15		1144	99%	AF150794.1
SA3	*Methylocystis echinoides*	*S. fallax*	787	96%	AJ458502
	*Methylosinus* sp. B3R		603	98%	AB636301
SM4	*Methylomonas sp.* R-45372	*S. magellanicum* 4	941	94%	FR798959.1
	*Methylomonas sp.* LC 1		1079	99%	DQ119049.1

## Discussion

Enriched cultures of microorganisms are an essential step in the production of inoculum of different organisms for biotechnology and bioengineering. In our studies, we showed an ability of the endophytic bacterial community to be cultured only at 20 and 30°C (Figure 1, 3). Most of methanotrophs hitherto isolated were classified as mesophilic (Hanson and Hanson [1996]). There were registered isolates belonging to the psychrophile group, whose optimum growth was reported at 3.5-10°C and a slight growth at 20°C (Omel’chenko et al. Omel'chenko et al. [1993]; Trotsenko and Khmelenina [2005]). Methanotrophic microorganisms belonging to the thermophilic/thermostable group, whose optimum growth was determined in the range of 40 - 65°C, have been identified (Trotsenko et al. [2009]). It is worth noting that the psychrophilic methanotrophs do not occur in the temperate climate zone, subtropical and tropical zones.

The use of the enrichment culture of endophytes allowed determination of the length of the adaptation phase (Figure 2) and the rate of growth at different concentrations of methane, which may be a forecast in the use of these cultures for biotechnology and significantly reduce the time to adapt in successive cycles of life. Analysis of methanotrophic activity showed that optimal oxidation of methane was found below 10% v/v of the initial methane concentration and at 30°C. Next, the culture was run at higher availability of methane from 3.167 to 3.678 μM CH_4_ per ml liquid culture and per day (Figure 3). If we apply the culture to pilot-scale bioreactors (200 dm^3^), we need to provide only from 0.633 mM to 0.736 mM CH_4_ per day for methanotrophic endophytes isolated from different species of *Sphagnum* sp. As a result, we believe that the culture of methanotrophic endophytes will be promising for environmental biotechnology. Besides, methane, which is the sole source of carbon and energy for these bacteria, is often released as a waste product into the atmosphere; for instance, the use of methane recovered as a biogas from waste deposits for energy purposes could become unprofitable.

The composition of the bacterial community possible to culture at 30°C and 10% of CH_4_ was identified (Table 3). The results show that the SF1 belongs to the genus *Methylocystis* and SM2 and SA3 to *Methylomonas*. The most diverse community is SA3, which consists of cultured strains belonging to the genera: *Methylosinus, Methylocystis,* and *Methylomonas.* (Steenbergh et al. [2010]) suggested different life strategies for these two groups of methanotrophic bacteria type I and II. In their view, type I methanotrophs have lower initial cell numbers in combination with the fast growth rate, which is in agreement with an R-type life strategy, investing in reproduction (Steenbergh et al. [2010]) instantaneous upon the presence of favorable conditions. In contrast, type II methanotrophs have higher initial cell numbers and slower growth. This situation is referred to as a K-type life strategy, connected with investing in survival and longevity (Andrews and Harris [1986]; Golovlev [2001]; Noll et al. [2005]). The communities isolated from *Sphagnum* sp. represent both the R and K strategy of life. Thus, they can be used in different environmental conditions, especially SA3, which comprises a mixture of type I and II methanotrophs.

An important element of this work was the culture of the methanotrophic communities isolated from *Sphagnum* sp. plants and recognition of their properties, because essentially, a consortium achieves the same role as does a genetically manipulated (or engineered) strain, but with superior stability. Genetically engineered strains are invariably both physiologically unbalanced and fastidious. (Hamer [2010]). As a matter of fact, methanotrophs are not able to exist in the form of pure cultures, because these are their unnatural conditions of life. For growth, methanotrophs require associated microorganisms necessary for their normal functioning (Hoefman et al. [2010]). This hypothesis was confirmed by FISH analysis of the cultured methanotrophic endophytes (Figure 5). Very numerous associated non-methanotrophic bacteria (dark blue cells) were observed, which probable support the growth of methanotrophic bacteria in the tested conditions (Eller et al. [2001]). In the literature, more effective neutralization of harmful factors was confirmed, when strains formed a mutualistic type of correlations in biofilm or aggregates (Tay et al. [2004]). The use of a consortium of micro-organisms, which exhibit different genomic and phenotypic diversity and the ability to aggregate, shall ensure much more efficient purification of the environment for several reasons: 1) bacterial cells are more resistant to toxic factors, 2) physiological diversity of microorganisms leads to increased degradation of contaminants in a single bioreactor and 3) separating of aggregated cells from a mixture is easy to perform (Tay et al. [2004]).

Negative contrast imaging of the cultured endophyte community by transmission microscopy showed the existence of structures facilitating communication between cells. These crystalline-like structures have been described as connecting lines referred to as nanopods (Shetty et al. [2011], Figure 4). So far, these organelles, which are used to transport the virulence factors over long distances, have been described only for pathogens such as *Pseudomonas aeruginosa* and *Legionella pneumophila* (Bomberger et al. [2009]; Ellis and Kuehn [2010]). The structure of nanopods of non-pathogenic bacteria has been identified for CS1 *Delftia* sp. 4 isolated from Wisconsin soil contaminated with polycyclic hydrocarbons (Shetty et al. [2011]). Nanopods have a crystalline-like outer surface and exhibit inner structures with varied (from spherical to spiral) morphology. Furthermore, using the three-dimensional electron cryotomography technique, (Shetty et al. [2011]) demonstrated that nanopods have tubular architecture, unlike the linear, filamentous construction characteristic of flagella or pili. Nanopods of *Delftia* sp. 4 projecting from cell surfaces were abundant and often spanned the space between neighboring bacteria, as for the cell surfaces of the methanotrophic endophytes (Figure 3). (Shetty et al. [2011]) suggested that formation of nanopods and the S-layer by bacterial cells are partially dependent on each other. The presence of the S-layer has been confirmed in many species of methanotrophic, for example halotolerant or thermotolerant, as well as non-halophilic strains (Khmelenina et al. [1999]; Khmelenina et al. [2010]). Our studies also indicate that methanotrophic endophytes have the S-layer, which, in contrast to carbohydrates, was observed in the presence of ruthenium red (Khmelenina et al. [1999]).

There are data indicating that bacteria able to produce nanopods have diverse lifestyles, for instance *Delftia* sp. Cs1-4, *D. acidovorans* SPH1, and *A. delafieldii*, which are free-living soil bacteria. In contrast, *A. avenae* subsp. *avenae* ATCC 19860, *A. avenae* subsp. *Citrulli*, and *V. eiseniae* are biotrophs and they live associated with eukaryotic organisms. Nanopods are produced by plant pathogens and earthworm symbionts (*V. eiseniae* EF01-2), which inhabit environments of decomposing organic material (Pinel et al. [2008]). The existence of these structures in methanotrophic endophytes is probable. Notably, considering the lifestyles of these microorganisms as well as the ecological niche (high decomposition of organic matter) from which they were isolated, it will be interesting to determine how, or if, bacteria tailor nanopods for unique functions in methanotrophs and recognize their structure.

In summary, contemporary physical/chemical treatment methods for decreasing the CH_4_ concentration such as activated carbon adsorption or incineration are either inefficient or costly at the low concentrations typically found in emissions from waste treatment and animal farming (López et al. [2013]). Therefore, properly tailored bioengineering can become a low-cost and environmentally friendly alternative to the physical/chemical methods for the abatement of CH_4_ (López et al. [2013]). We have described a unique methanotrophic community, which can be a good example for future microbial biotechnology, which has not been quite recognized until recently as far as natural environmental processes were concerned. The proposal presented can indicate the scope of ideas and concepts in process-oriented environmental biotechnology.

## Competing interests

The authors declare that they have no competing interests.

## Authors’ contributions

ZS have made substantial contributions to conception and design, or acquisition of data, or analysis and interpretation of data. AK carried out the molecular genetic studies, participated in the sequence alignment, drafted the manuscript, in the design of the study and performed the statistical analysis. Both authors read and approved the final manuscript.
